# Sex-specific public health data: analyzing the arboviral impact on women in Brazil

**DOI:** 10.11606/s1518-8787.2025059006235

**Published:** 2025-06-11

**Authors:** Brena F. Sena, Danyelly Bruneska Gondim Martins, Carmen Simone Grilo Diniz, José Luiz Lima

**Affiliations:** I Universidade Federal de Pernambuco Instituto Keizo Asami Recife PE Brazil Universidade Federal de Pernambuco. Instituto Keizo Asami. Recife, PE, Brazil; II Universidade Federal de Pernambuco Instituto Keizo Asami Departamento de Bioquímica Recife PE Brazil Universidade Federal de Pernambuco. Instituto Keizo Asami. Departamento de Bioquímica. Recife, PE, Brazil; III Universidade de São Paulo Faculdade de Saúde Pública Departamento de Saúde, Ciclos de Vida e Sociedade São Paulo SP Brazil Universidade de São Paulo. Faculdade de Saúde Pública. Departamento de Saúde, Ciclos de Vida e Sociedade. São Paulo, SP, Brazil

**Keywords:** Arboviral Infections, Women’s Health, Maternal Health, Sex-Specific Data, Geospatial Analysis, Brazil

## Abstract

**OBJECTIVE:**

To evaluate the differential impact of arboviral infections, specifically dengue virus, chikungunya virus, and Zika virus, on women in Brazil, with a focus on sex- and age-disaggregated analyses.

**METHODS:**

A comprehensive epidemiological and geospatial data analysis was conducted utilizing data from Brazil’s national health data system, including the disease notification system (*Sistema de Informação de Agravos de Notificação*) and mortality information system (*Sistema de Informação sobre Mortalidade*), covering national and municipal level data. Arboviral case notification rates were analyzed using generalized linear mixed models with negative binomial regression, stratified by sex, age group, and year. Geospatial visualizations mapped the case rate distribution highlighting the top municipalities with the most female case rate and hospitalizations rate. All analyses were implemented in the statistical software R.

**RESULTS:**

Significant sex- and age-stratified differences were observed in the arbovirus notification rates for dengue virus, chikungunya virus, and Zika virus over the past seven years, with consistently higher rates among women compared to men. Stratified analyses revealed that females aged 20–59 years, particularly those of reproductive age, bore a disproportionately higher burden across all three viruses. The low serotyping resolution for the dengue virus constrained further granular analysis, particularly for severe outcomes such as hospitalizations and mortality based on dengue serotype.

**CONCLUSION:**

Sex- and age-disaggregated epidemiological surveillance is critical to inform public health policies and interventions targeting arboviral diseases. This study underscores the necessity of incorporating sex-specific data analyses to optimize responses for vulnerable female populations. Geospatial visualizations reveal infection hotspots, providing actionable insights for region-specific interventions to improve health outcomes in Brazil.

## INTRODUCTION

In the early 2000s, the United Nations Millennium Development Goals (MDGs) established a global agenda with an emphasis on improving maternal health. Brazil, however, struggled to meet MDG 5, which aimed to reduce maternal mortality by 2015^1^. This shortfall underscored the urgency of addressing gender-based inequities, a priority that was carried forward in the United Nations Sustainable Development Goals (SDGs) launched in 2016. Among these, SDG Goal 5 further emphasized the promotion of gender equality and women’s empowerment, yet progress in Brazil remains slow, especially regarding gender-specific objectives, with the 2030 SDGs goal deadline looming^2,3^.

The Covid-19 pandemic, which began in 2020, intensified existing vulnerabilities, disproportionately affecting Brazilian women^4-7^. The pandemic intensified domestic violence, expanded the burden of unpaid domestic labor, and hindered women’s career advancement^8,9^. Brazilian women, particularly from low-income groups, faced restricted healthcare access, compounded by physical and mental health challenges^5,7^. Despite higher average educational attainment among women in Brazil compared to men in all age groups, stark disparities persist in rural compared to urban areas, notably in secondary school completion rates^8,9^.

Concurrent with these socio-economic challenges, Brazil faces the recurring public health burden of arboviral infections, with dengue virus (DENV), chikungunya virus (CHIKV), and Zika virus (ZIKV) contributing to morbidity and mortality. Notably, 2024 marked a peak year for arboviral infection cases, with 6.5 million cases of DENV, 250,000 cases of CHIKV, and 6,300 cases of ZIKV reported by mid-year^10,11^. Importantly, these infections pose a heightened risk to women, with broader implications for maternal health^12-14^. For instance, DENV infections have been shown to triple the risk of maternal mortality^15,16^, whereas ZIKV infection is linked to congenital Zika syndrome, including microcephaly^17,18^. Co-infections of DENV, CHIKV, and ZIKV, and rare triple infections, present additional health risks, particularly among pregnant women^19,20,21^.

Epidemiological data from Rio de Janeiro revealed that nearly 60% of arboviral cases occurred among women^22^. However, the current reporting mechanisms in Brazil, largely based on static Ministry of Health reports, often lack the timeliness and granularity needed for effective, localized public health responses and insights. The newly developed live dashboards for real-time data dissemination represent a step forward in improving the public health infrastructure; however, challenges remain in integrating the diverse datasets part of the wider DATASUS system and in the spatial representation of arboviral data across geographic regions^23,24^.

This study aims to address these gaps by evaluating the sex- and age-specific burden of arboviral infections in Brazil. Our analysis leverages the robust data architecture of Brazil’s DATASUS system, offering open access to epidemiological data at both the municipal and national levels^11,25^. By incorporating advanced geospatial visualizations at the municipal level and disaggregating arboviral case data by sex and age, we aim to identify vulnerable populations and provide actionable insights for public health interventions^23,24^.

## METHODS

This study analyzed arbovirus case notifications for dengue (DENV), chikungunya (CHIKV), and Zika (ZIKV) between 2017 and 2024, stratified by sex, age group, and year. The analysis involved a two-step statistical approach combining inferential statistics and regression modeling to explore trends and relationships across key variables.

### Two-Proportion Z-tests

To evaluate sex-based differences in case notifications, two-proportion z-tests were conducted separately for each virus and each year in the study period. This test compared the proportions of cases between males and females, generating z-scores and corresponding p-values. The statistical significance threshold was set at p < 0.05. These tests helped identify whether there were statistically significant differences in arbovirus notifications between the sexes over time. Additionally, for each virus-year pair, the percentage of cases among males and females was calculated to support the interpretation of the results.

### Negative Binomial Generalized Linear Model (NB-GLM)

Given the count nature of the dependent variable (arbovirus notifications), a Negative Binomial Generalized Linear Model (NB-GLM) was applied to account for potential overdispersion in the data^32^. Separate models were fitted for DENV, CHIKV, and ZIKV, with the number of confirmed cases as the dependent variable and sex, age group, and year as independent variables. Age was categorized into the following groups: 1–4, 5–9, 10–14, 15–19, 20–39, 40–59, 60–64, 65–69, 70–79, and 80+ years. The model structure was defined as follows:


 cases ∼ sex + age group + year

Where “cases” represents the number of confirmed notifications for each virus. The regression coefficients were exponentiated to yield incidence rate ratios (IRRs), which provide insights into the relative risk of arbovirus notification for each sex, age group, and year compared to the reference categories (e.g., females, age group 20–39, and the baseline year 2017).

### Model Diagnostics and Fit

The performance and fit of the models were evaluated using the Akaike Information Criterion (AIC), Bayesian Information Criterion (BIC), deviance, and pseudo-R-squared values. These diagnostics were used to assess the goodness-of-fit and relative performance of the models across the three arboviruses, providing a rigorous basis for determining which model best explained the variability in case notifications.

### Data Source

The case notification data for DENV, CHIKV, and ZIKV were sourced from Brazil’s national health information system, *Sistema de Informação de Agravos de Notificação* (SINAN). This database provides confirmed arbovirus notifications at the municipal and national levels. Data wrangling and preparation were conducted using the “dplyr” package in R, and NB-GLMs were fitted using the “MASS” package.

### Visualization and Reporting

**Figure 1:**
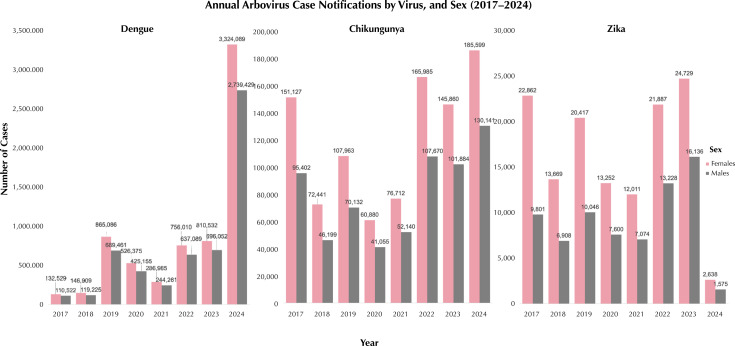
Annual arbovirus case notifications by virus (dengue, chikungunya, and zika), and sex (2017–2024).

**Figure 2:**
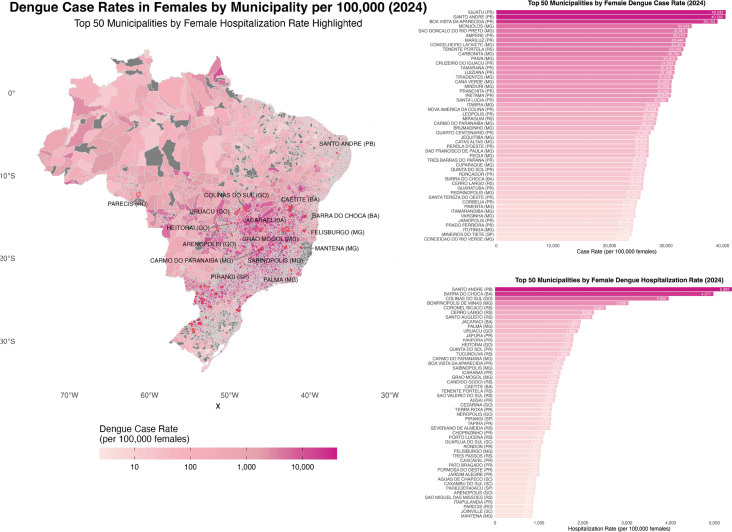
Dengue case rates in females by municipality per 100,000 and top 50 municipalities with highest dengue case rate and hospitalization rates in females. Brazil, 2024.

All visualizations were generated in R using “ggplot2” and “dplyr”. Stacked bar charts were created to illustrate annual case notifications by virus and sex (Figure 1), while additional visualizations stratified by age group highlighted temporal trends. For geospatial visualizations, maps were created to display the distribution of female arboviral cases, especially DENV cases across Brazilian municipalities (Figure 2), with a focus on identifying the top municipalities with the highest number of DENV case rates and DENV-related hospitalizations rates among females^33^. To ensure comparability across municipalities with varying population sizes, female DENV case and hospitalization counts were normalized by dividing the number of confirmed DENV cases and hospitalizations in females by the female population in each municipality and multiplying by 100,000 (Figure 2). Female population estimates were sourced from the 2022 IBGE Brazilian census data and matched to corresponding municipality codes. These normalized rates were then visualized in choropleth maps and ranked bar charts to identify municipalities with the highest burden of DENV among females.

### Software

All analyses were performed using R (version 4.3.0) in RStudio (version 2023.06.0+421), with specific packages used for data manipulation (“dplyr”), statistical modeling (“MASS”), and visualization (“ggplot2”).

### Ethical Aspects

This study did not need approval from the Ethics Committee on Research on Human Beings due to the use of de-identified and publicly available data.

## RESULTS

Our analysis of DENV, CHIKV, and ZIKV cases from 2017 to 2024 revealed significant sex- and age-specific differences in the case distributions across Brazil. Using NB-GLMs, we achieved robust model performance, with explanatory power for each virus (Tables 1 and 2). The DENV model had a pseudo-R-squared of 0.9859, explaining 98.59% of the variance, while the CHIKV and ZIKV models demonstrated similarly strong fits with pseudo-R-squared values of 0.9776 and 0.9538, respectively (Table 2).

**Table 2:** Model fit statistics for dengue, chikungunya, and zika virus, negative binomial Generalized Linear Model (GLM).

Statistic	Dengue	Chikungunya	Zika Virus
AIC	3,914,803	2,906,413	2,313,628
BIC	3,983,857	2,969,822	2,377,038
Residual Deviance	199,332	177,3825	176,4979
Null Deviance	14,133,530	7,933,322	3,823,939
Pseudo R-squared	0.9859	0.9776	0.9538
Theta (Dispersion)	33.82 (SE = 3.40)	33.18 (SE = 3.57)	16.60 (SE = 1.81)
2 x Log-likelihood	-3,872,803	-2,866,413	-2,273,628
Number of observations	198	176	176
Degrees of Freedom	178	157	157
Fisher Scoring Iterations	1	1	1

Note: Model fit statistics for Dengue, Chikungunya, and Zika Virus, including Akaike Information Criterion (AIC), Bayesian Information Criterion (BIC), and Pseudo R-squared values. Theta represents the dispersion parameter with the standard error (SE).

The model selection criteria, including AIC and BIC, further supported the strength of the models, with lower values indicating better fit. The AIC values for DENV, CHIKV, and ZIKV were 3914.803, 2906.413, and 2313.628, respectively, while the BIC values were slightly higher at 3983.857, 2969.822, and 2377.038. Residual deviance values for DENV (199.332), CHIKV (177.3825), and ZIKV (176.4979) were markedly lower than their respective null deviances, confirming the strength of NB-GLM in reducing unexplained variance. These diagnostics, along with the high pseudo-R-squared values and substantial reductions in deviance, confirm the NB-GLMs’ capacity to accurately capture the complexity of arboviral case distributions across demographic groups in Brazil.

In terms of sex-based disparities, females consistently exhibited higher case counts compared to men across all three arboviruses (Figure 1 and Table 1). The sex coefficients were negative and highly significant (p < 0.001), with the strongest effect observed for ZIKV (-0.32728) and the weakest for DENV (-0.15088), indicating that being female is associated with a greater risk of arboviral infection. This finding aligns with previous studies^22^, which also reported increased susceptibility and exposure to arboviruses among women. For DENV, females accounted for over half of the cases each year, ranging from 53.79% in 2023 to 55.65% in 2019. Statistically significant sex differences were found across all years except 2017, with z-tests indicating significantly higher female case rates in most years (p < 0.001). For CHIKV, females consistently represented the majority of cases annually, peaking at 61.46% in 2017, with significant differences observed in all years except 2020. ZIKV showed the most pronounced sex disparity, with females comprising a large majority of cases each year, peaking at 70.00% in 2017. Statistically significant differences favoring females were observed in all years except 2018.

**Table 1:** Negative binomial Generalized Linear Model (GLM) statistics for dengue, chikungunya, and zika virus by sex, age group, and year.

Variable	DENV coefficient	DENV Std Error	DENV p-value	CHIKV coefficient	CHIKV Std Error	CHIKV p-value	ZIKV coefficient	ZIKV Std Error	ZIKV p-value
Intercept	9.287	0.054	< 2e-16 ***	7.525	0.057	< 2e-16 ***	6.474	0.082	< 2e-16 ***
Sex (Male vs. Female)	-0.151	0.024	7.09e-10 ***	-0.275	0.026	< 2e-16 ***	-0.327	0.037	< 2e-16 ***
Age Group (years)									
Age < 1 year	-	-	-	-	-	-	-	-	-
Age 1–4 years	0.749	0.057	< 2e-16 ***	0.537	0.062	< 2e-16 ***	0.253	0.088	0.0041 **
Age 5–9 years	1.368	0.057	< 2e-16 ***	1.157	0.062	< 2e-16 ***	0.496	0.088	1.76e-08 ***
Age 10–14 years	1.657	0.057	< 2e-16 ***	1.420	0.062	< 2e-16 ***	0.576	0.088	5.88e-11 ***
Age 15–19 years	1.883	0.057	< 2e-16 ***	1.601	0.062	< 2e-16 ***	0.782	0.088	< 2e-16 ***
Age 20–39 years	3.297	0.057	< 2e-16 ***	3.275	0.062	< 2e-16 ***	2.351	0.088	< 2e-16 ***
Age 40–59 years	2.916	0.057	< 2e-16 ***	3.122	0.062	< 2e-16 ***	1.719	0.088	< 2e-16 ***
Age 60–64 years	1.045	0.057	< 2e-16 ***	1.327	0.062	< 2e-16 ***	-0.319	0.089	0.0003 ***
Age 65–69 years	0.738	0.057	< 2e-16 ***	1.068	0.062	< 2e-16 ***	-0.672	0.089	4.35e-14 ***
Age 70–79 years	0.839	0.057	< 2e-16 ***	1.209	0.062	< 2e-16 ***	-0.575	0.089	9.77e-11 ***
Age 80+ years	-0.132	0.058	0.0214 *	0.271	0.062	1.32e-05 ***	-1.552	0.091	< 2e-16 ***
Year									
Year 2017	-1.800	0.052	< 2e-16 ***	-	-	-	-	-	-
Year 2018	-1.713	0.052	< 2e-16 ***	-0.749	0.053	< 2e-16 ***	-0.381	0.076	4.81e-07 ***
Year 2019	0.029	0.052	0.5703	-0.346	0.053	4.66e-11 ***	-0.036	0.075	0.6358
Year 2020	-0.455	0.052	< 2e-16 ***	-0.929	0.053	< 2e-16 ***	-0.362	0.076	1.72e-06 ***
Year 2021	-1.017	0.052	< 2e-16 ***	-0.662	0.053	< 2e-16 ***	-0.442	0.076	5.17e-09 ***
Year 2022	-0.021	0.052	0.6790	0.105	0.053	0.0449 *	0.251	0.075	0.0009 ***
Year 2023	-0.049	0.052	0.3428	0.013	0.053	0.8076	-0.209	0.075	0.0057 **
Year 2024	1.531	0.052	< 2e-16 ***	0.391	0.053	9.67e-14 ***	0.046	0.075	0.5458

Note: Estimated coefficients, standard (Std) errors, and p-values for the predictors in the models for the Dengue virus (DENV), Chikungunya virus (CHIKV), and Zika virus (ZIKV). Significance levels are indicated by asterisks: ***p < 0.001, **p < 0.01, *p < 0.05.

When analyzing the data by age group, a consistent pattern emerged across all three viruses. The 20–39 and 40–59 age groups had the highest relative number of cases compared with the reference group (0–4 years). For example, in the DENV model, individuals aged 20–39 years had a coefficient of 3.297 (p < 0.001), indicating a significantly higher number of cases in this age group. This pattern was similarly observed in the CHIKV and ZIKV models although the magnitude of the effect varied slightly between the viruses. These results may reflect behavioral, occupational, or biological factors.

Temporal trends varied by virus, with the early years (2017–2021) showing lower DENV case counts relative to 2024, which saw a resurgence. Specifically, the DENV model showed significant negative coefficients for years like 2017 (-1.800) and 2018 (-1.713), both with p-values < 0.001, indicating lower case counts compared to 2024. CHIKV also saw a significant rise in 2024 (coefficient = 0.391, p < 0.001), marking an increase in notifications. ZIKV trends were less uniform but displayed an increase in 2024, reflecting variable yearly case numbers across the study period.

The two-proportion z-tests further substantiated these sex-based findings. For DENV, significant sex differences (p < 0.001) were observed in most years, with females having consistently higher case rates than males, except in 2017. CHIKV results showed significant sex-based differences in every year except 2020, with females, especially in the reproductive age group, reporting higher case counts. ZIKV exhibited significant sex differences in all years except 2018, with females showing consistently higher case numbers, particularly among those of reproductive age.

For CHIKV, we found significant sex-based differences in every year from 2017 to 2024, except for 2020. Females consistently had higher case counts, especially within the reproductive age group (15–49 years). The highest case numbers occurred in 2017 and 2022, with 10,882 cases in females compared to 8,091 in males in 2017, and 9,501 cases in females versus 7,307 in males in 2022. Case numbers declined after 2017, reaching their lowest point in 2020, but fluctuated in subsequent years. These trends may reflect various factors, including differences in exposure, healthcare-seeking behavior, or biological susceptibility to CHIKV between sexes.

For ZIKV, statistically significant sex differences (p < 0.001) were observed in most years, except for 2018, where the disparity was less pronounced. In every year from 2017 to 2024, females in the reproductive age group consistently had higher ZIKV case numbers compared with males. The year 2016 had the highest case numbers for both sexes, with 16,682 cases in females compared to 7,856 in males. These findings support the hypothesis that females, particularly those of reproductive age, may be more affected by ZIKV, possibly due to increased healthcare engagement during pregnancy or biological vulnerability to the virus.

These findings emphasize the need for targeted, sex-specific public health interventions, particularly for women, who bear a higher burden of arboviral infections. The disparities observed suggest that a combination of factors—biological, socioeconomic, and behavioral—may drive these sex differences. For instance, socioeconomic challenges in urban areas such as Recife^21^, alongside national disparities in school enrollment^26,28^, labor force participation, and female political representation, may intensify the impact of arboviruses on women, especially those facing additional social vulnerabilities.

## DISCUSSION

Across all three viruses (DENV, CHIKV, and ZIKV), the models consistently showed that males had significantly lower reported case counts than females. This persistent difference suggests that women are disproportionately affected by arboviruses. A critical consideration is that the reproductive age range for women (roughly 15–49 years) aligns with the age groups showing the highest infection rates. This overlap may reflect a combination of biological, immunological, and socio-behavioral factors, including healthcare-seeking behaviors and differing exposure risks. Women may be more exposed to vectors due to roles in domestic responsibilities, such as proximity to breeding sites like water storage areas, or participation in informal work sectors that increase contact with mosquitoes. Additionally, pregnant women are often subjected to more frequent testing due to the known risks of ZIKV to fetal health, potentially inflating case counts in this group. Hormonal changes, particularly during pregnancy, may also increase susceptibility to arboviruses like ZIKV.

The 20–39 and 40–59 age groups, which encompass women’s reproductive years, exhibited the highest number of cases across all viruses, highlighting a critical public health concern. Women in this age range are often involved in caregiving, outdoor activities, or employment that may increase mosquito exposure, while also facing increased biological vulnerability related to reproductive or hormonal factors. This pattern remained consistent across all three viruses and warrants further exploration.

The models also demonstrated temporal variations, with certain years, notably 2024, showing an increase in reported cases. Despite these fluctuations, the higher infection rates among women remained constant, underscoring the need for public health interventions that account for sex-specific vulnerabilities, particularly for women of reproductive age. Therefore, public health efforts should prioritize protecting this group, which is disproportionately affected by arboviral infections.

To further contextualize these disparities, geospatial maps and ranked bar charts were developed to visualize municipal-level differences in female DENV case and hospitalization rates (Figure 2). By normalizing case counts per 100,000 females using 2022 IBGE census estimates, these visualizations allowed for equitable comparisons across municipalities with widely varying population sizes, highlighting areas with disproportionate disease burdens among females that may otherwise go unnoticed.

Addressing these sex-based differences is crucial for shaping public health strategies^35^. Effective interventions must go beyond monitoring to refine disease prevention, treatment, and support programs tailored to the needs of women. Further research is needed to explore how age, comorbidities, and behavioral factors influence disease severity, morbidity, and mortality among men and women. Despite the significant burden of DENV among women, current vaccine strategies remain limited in scope. The only currently approved vaccines for DENV in Brazil, are either restricted to individuals who are dengue-naïve or require prior infection confirmation, and are not recommended for use in pregnant women due to insufficient safety data. This leaves a critical gap in prevention efforts for a population that is disproportionately affected and at heighted risk of severe outcomes, including maternal mortality. This disparity highlights the importance of considering sex-based differences in the spread and impact of these diseases when planning public health strategies^15^. To better address the unique burden women face, collecting sex-disaggregated data is essential, as are targeted intervention strategies that allocate financial and social resources^32^ to support women more effectively^13,14^.

Brazil’s data infrastructure, while publicly accessible, presents challenges in integrating data across databases and at the municipal level, especially from databases like SINAN, SIM, and *Instituto Brasileiro de Geografia e Estatística* (IBGE)^34^. Inconsistencies in municipal coding and potential data capture biases hinder a comprehensive understanding of arboviral disease burdens. Furthermore, the lack of ICD-10 codes for CHIKV- and ZIKV-related mortality complicates accurate mortality tracking, in contrast to DENV, where clearer reporting mechanisms exist. Although mortality data are available through SIM, they are fragmented, highlighting the need for systematic and connected data, including demographic information, medical history, and laboratory results.

Another significant limitation is the potential for the misclassification of CHIKV and ZIKV cases as DENV due to hierarchical reporting protocols, likely introducing biases in the case evaluation. Combined with the high prevalence of asymptomatic and unreported arboviral cases, particularly for DENV, these factors skew our understanding of disease trends. Public health efforts should prioritize the accurate serotyping of DENV cases, especially among hospitalized individuals, women, and pregnant women^27^, and invest in genotyping to detect and monitor the circulation of new or more virulent strains.

Improving data collection systems to include comorbidities (e.g. cardiovascular risks^29^, pregnancy-related complications like preeclampsia^29^), nutritional status, immune function^30^, and body mass index (BMI) is essential. Data on symptoms, disease severity, and vaccination status (particularly for DENV) should be systematically incorporated into public health databases, along with diagnostic results for arboviral infections.

The limitations of our study primarily arise from the constraints of publicly available data. A unified approach for geospatial data standardization and management is also crucial for accurately mapping infection trends and guiding intervention efforts, especially during public health crises. As of 2024, Brazil is experiencing an unprecedented spike in DENV cases, alongside significant reports of CHIKV and ZIKV infections. The extreme weather witnessed this year intensified by climate change are fueling the spread of arboviruses, and the link between rising temperatures and increased vector activity has significant public health implications. These conditions, along with advancements in data visualization tools such as interactive dashboards and geospatial maps, offer an opportunity to enhance the monitoring and analysis of infection rates by sex and other demographic factors across different regions and timeframes. Such tools can provide critical insights for developing more precise and effective public health responses.

## CONCLUSIONS

Addressing the unique health challenges posed by arboviral infections in Brazil, particularly for women, requires a comprehensive strategy that accounts for sex-based and age-based considerations. This approach should involve innovative data collection methods and advanced analytical techniques that focus on sex-specific and age-related impacts. Consistent and detailed arboviral data collection, along with geospatial information, will enhance analysis and visualizations, supporting informed policymaking and improving public health outcomes for specific populations.

Our analysis of DENV, CHIKV, and ZIKV cases using Negative Binomial GLM models revealed significant demographic and temporal patterns. These findings underscore the need for public health interventions targeted at vulnerable populations, particularly women in reproductive age groups, who are disproportionately affected by these viruses. The use of normalized, sex-specific case rates enabled more accurate identification of municipalities with disproportionately high burdens of disease among women, supporting targeted, regionally sensitive public health interventions. Public health strategies must prioritize these groups to mitigate the impact of arboviruses in Brazil.

To fully understand the societal and health impacts of arboviruses, particularly on women, it is essential to extend data collection over a longer period, ideally 10–15 years. Such extended data collection should include sex-specific indicators and age-based approaches to illuminate the heightened exposure risks for women and potential comorbidities. The use of normalized, sex-specific case rates enabled more accurate identification of municipalities with disproportionately high burdens of disease among women, supporting targeted, regionally sensitive public health interventions. Furthermore, social and political factors contributing to increased exposure or risk should be examined, as these insights will guide the allocation of resources and the provision of care more effectively.

Longitudinal studies focused on both affected and unaffected women, as well as pregnant women, are critical to understanding the long-term effects of arboviruses on women’s health, including maternal and child health outcomes. Without a focused approach that emphasizes sex and age, the true scope of the impact of arboviral infections on women will remain obscured, which could undermine efforts to address these public health challenges effectively.

This study highlights the intersection of gender, age, and socioeconomic factors in contributing to arbovirus infection rates in Brazil. Further research on sex- and age-based disparities in arbovirus rates and outcomes will support efforts to improve women’s health and help close the gender and sex gaps in public health, both in Brazil and globally.

**Table 3:** Two-proportion z-tests with p-values for differences in dengue, chikungunya, and zika virus notification case rates by sex (2017–2024).

Year	Virus	Female Cases	Male Cases	Total Female %	Total Male %	Z-Statistic	p-value	Significance
2017	DENV	132,529	110,522	54.52	45.47	1.919	0.0549	NS
2018	DENV	146,909	119,225	55.20	44.79	-5.077	4.47e-07	***
2019	DENV	865,086	689,461	55.65	44.35	-24.856	2.22e-136	***
2020	DENV	526,375	425,155	55.32	44.68	-12.217	2.52e-34	***
2021	DENV	286,985	244,261	54.02	45.97	10.443	1.57e-25	***
2022	DENV	756,010	637,089	54.26	45.73	11.334	8.82e-30	***
2023	DENV	810,532	696,052	53.79	46.20	24.164	5.29e-129	***
2024	DENV	3,414,971	2,820,664	54.76	45.23	-3.249	1.15e-03	**
2017	CHIKV	152,127	95,402	61.46	38.54	-15.945	3.04e-57	***
2018	CHIKV	72,441	46,199	61.06	38.94	-7.648	2.04e-14	***
2019	CHIKV	107,963	70,132	60.62	39.38	-5.558	2.72e-06	***
2020	CHIKV	60,880	41,055	59.72	40.27	1.936	0.0528	NS
2021	CHIKV	76,712	52,140	59.53	40.46	3.642	2.70e-04	***
2022	CHIKV	165,985	107,670	60.65	39.34	-7.523	5.33e-14	***
2023	CHIKV	145,860	101,884	58.87	41.12	12.539	4.54e-36	***
2024	CHIKV	198,513	138,845	58.84	41.15	15.554	1.49e-54	***
2017	ZIKV	22,862	9,801	70.00	30.00	48.963	6.98e-50	***
2018	ZIKV	13,669	6,908	66.42	33.58	37.986	5.61e-01	NS
2019	ZIKV	20,417	10,046	67.03	32.97	56.300	2.90e-03	**
2020	ZIKV	13,252	7,600	63.55	36.45	36.674	4.77e-17	***
2021	ZIKV	12,011	7,074	62.94	37.06	31.243	6.41e-23	***
2022	ZIKV	21,887	13,228	62.31	37.69	36.352	2.79e-58	***
2023	ZIKV	24,729	16,136	60.51	39.48	22.277	2.25e-144	***
2024	ZIKV	2,638	1,575	62.61	37.38	4.998	5.79e-07	***

Note: This table presents the results of two-proportion Z-tests, showing annual case counts, percentage distribution by sex, Z-statistic, and p-values for Dengue virus (DENV), Chikungunya virus (CHIKV), and Zika virus (ZIKV) from 2017 to 2024. Significance levels are indicated by asterisks: ***p < 0.001, **p < 0.01, *p < 0.05. NS denotes “not significant.”
